# CdSe/ZnS quantum dot encapsulated MoS_2_ phototransistor for enhanced radiation hardness

**DOI:** 10.1038/s41598-018-37902-y

**Published:** 2019-02-05

**Authors:** Jinwu Park, Geonwook Yoo, Junseok Heo

**Affiliations:** 1 Department of Electrical and Computer Engineering, Ajou University, Suwon, 16499 South Korea; 2 School of Electronic Engineering, Soongsil University, Seoul, 06938 South Korea

## Abstract

Notable progress achieved in studying MoS_2_ based phototransistors reveals the great potential to be applicable in various field of photodetectors, and to further expand it, a durability study of MoS_2_ phototransistors in harsh environments is highly required. Here, we investigate effects of gamma rays on the characteristics of MoS_2_ phototransistors and improve its radiation hardness by incorporating CdSe/ZnS quantum dots as an encapsulation layer. A 73.83% decrease in the photoresponsivity was observed after gamma ray irradiation of 400 Gy, and using a CYTOP and CdSe/ZnS quantum dot layer, the photoresponsivity was successfully retained at 75.16% on average after the gamma ray irradiation. Our results indicate that the CdSe/ZnS quantum dots having a high atomic number can be an effective encapsulation method to improve radiation hardness and thus to maintain the performance of the MoS_2_ phototransistor.

## Introduction

Two-dimensional materials such as transition metal dichalcogenides (TMD) have received considerable attention as a promising channel material for nanoelectronics devices^1–6^. Among a variety of TMD materials, molybdenum disulfide (MoS_2_) is the best known and is studied extensively. MoS_2_-based thin-film transistors have interesting electrical characteristics, such as high ON/OFF ratio (~10^8^) and high electron mobility (~200 cm^2^∙Vs^−1^)^1^. In addition, MoS_2_ is appropriate as an absorption layer of optoelectronic devices owing to its bandgap of 1.2–1.8 eV depending on its number of layers (bulk MoS_2_ has an indirect bandgap of 1.2 eV^7^ and single-layer MoS_2_ has a direct bandgap of 1.8 eV^8^), fast photo-switching (within 110 μs^9^), and absorption coefficient (*α* = 1–1.5 × 10^6^ cm^−1^ for single-layer MoS_2_^10^ and 0.1–0.6 × 10^6^ cm^−1^ for bulk MoS_2_^11^ in the wavelength of 500–1200 nm). Therefore, studies of using MoS_2_ as an absorption layer of an optoelectronic device have been actively conducted. It exhibits improved photoresponsivity^12–23^, wavelength selectivity^24,25^, and enhanced optical switching speed^9,26–28^. However, the research on the durability of MoS_2_ in harsh environments^9^ for use as photodetectors in various fields remains lacking.

Radiation (particularly ionizing radiation), one of the harsh environments, is encountered not only around nuclear reactors and particle accelerators but in outer space and high-altitude flights. Radiation has characteristics such as material permeability, atomic reaction capacity (nuclear reaction, excitation, scattering, etc.), biological actions, etc. Because of these features, radiation can be used in various fields such as medicine^29,30^ and agriculture^31,32^. However, gamma rays cause damage to the human body^33,34^ and materials^35–38^, resulting in the malfunction of instruments and devices. Thus, protecting devices from radiation is necessary.

Previous studies have only studied how radiation affects the structure of MoS_2_^37,38^. These studies alone cannot confirm that the performance reliability of the MoS_2_ phototransistor is guaranteed in places of high radiation. No studies have been conducted on the effect of radiation on the MoS_2_ phototransistor and the methods to prevent performance degradation by radiation. Therefore, it is necessary to study how the MoS_2_ phototransistor is affected by radiation and to prevent it.

In this study, we found that the photoresponsivity of the MoS_2_ phototransistor is significantly degraded by gamma rays. The degradation of the MoS_2_ phototransistor is due to the gamma rays causing a positive oxide charge on the gate dielectric. Therefore, we developed a MoS_2_ phototransistor with a CYTOP and CdSe/ZnS quantum dot (CYTOP/QD) layer as a gamma-ray-shielding layer, and confirmed that the CYTOP/QD layer efficiently prevented the degradation by gamma rays.

## Results and Discussion

**Figure 1:**
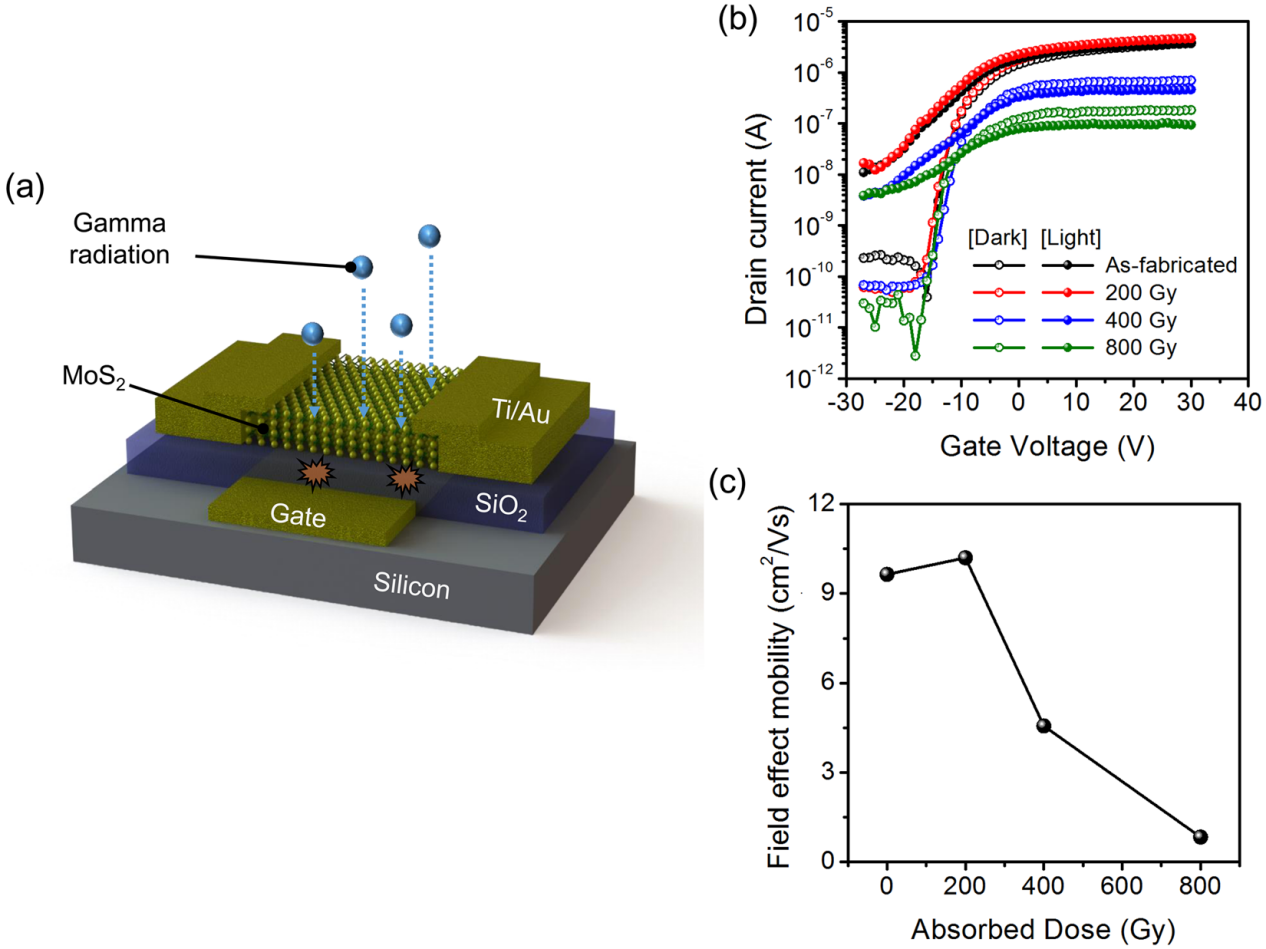
(**a**) Schematic of the bare MoS_2_ phototransistor. (**b**) Transfer curves in dark and photo-responses of the bare MoS_2_ phototransistor after irradiated by 0, 200, 400 and 800 Gy of gamma rays. (**c**) Variations of the field effect mobility in the bare MoS_2_ phototransistor after irradiated by different doses of gamma rays.

To investigate the degradation of the MoS_2_ phototransistor by gamma rays, we irradiated the MoS_2_ phototransistor with a gamma ray. Figure 1a shows the schematic diagram of the used MoS_2_ phototransistor. The MoS_2_ phototransistor was fabricated using MoS_2_ flakes exfoliated from bulk MoS_2_ by the conventional Scotch-tape method. For the gamma ray degradation test, a Co-60 was used as the gamma ray source, and an absorbed dose of 200, 400 and 800 Gy were irradiated to the MoS_2_ phototransistor. Figure 1b shows the transfer curves of the MoS_2_ phototransistor before and after gamma irradiation. We extracted the electrical characteristics from the transfer curves measured in the dark (open sphere in Fig. 1b). The threshold voltage (*V*_th_) and subthreshold swing did not show significant change even with increasing gamma ray dose. On the other hand, the on-current (*V*_GS_ > *V*_th_) apparently decreases. Figure 1c shows the change of the field effect mobility as the dose of gamma ray increases. The field effect mobility of the MoS_2_ phototransistor was 9.64 cm^2^/V∙s before irradiation of gamma rays, but slightly increased to 10.2 cm^2^/V∙s after irradiation of 200 Gy. When the absorbed dose was 400 Gy and 800 Gy, the field effect mobility was degraded to 4.56 cm^2^/V∙s and 0.836 cm^2^/V∙s, respectively. The field effect mobilities were estimated from the following equation^13,25^:

1 $${\mu }_{eff}=({g}_{m}\cdot L)/({C}_{OX}\cdot W\cdot {V}_{DS})$$

In Equation (1), $L$ is the channel length, $W$ is the channel width, ${C}_{{OX}}$ is the oxide capacitance, and ${V}_{{DS}}$ is the drain bias. These results imply that a small amount of gamma dose does not cause the degradation of the MoS_2_ phototransistor but affects the performance of the MoS_2_ phototransistor as the absorbed dose increases. As the absorbed dose increases, more atomic displacements are induced in MoS_2_ channel^38^, eventually impairing the MoS_2_ phototransistor permanently (see supplementary).

**Figure 2:**
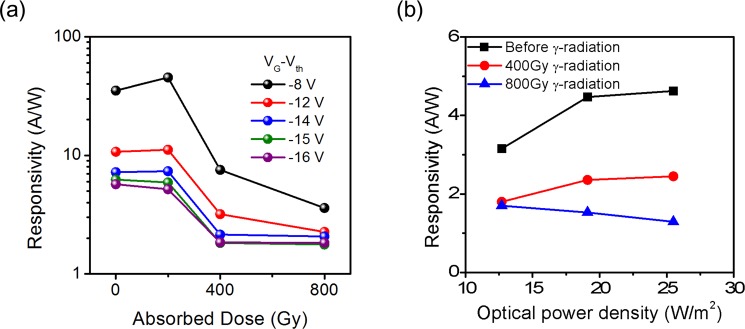
(**a**) Variations of the responsivities in the bare MoS_2_ phototransistor after irradiated by different doses of gamma rays. (**b**) Optical power density dependent responsivities of the bare MoS_2_ phototransistor after irradiated by 0, 400, and 800 Gy of gamma rays.

The responsivity according to absorbed dose showed similar trend to the field effect mobility. Figure 2a shows the change in responsivity as the absorbed dose increases when 19.1 W/m^2^ optical power density is incident. The responsivity is obtained using ${I}_{{ph}}/{P}_{{light}}$, where ${I}_{{ph}}$ is the difference between the drain current under illumination and the drain current in the dark, and ${P}_{{light}}$ is the optical power illuminated on the MoS_2_ channel. When 200 Gy of gamma rays were irradiated, there was very little change in responsivity, but rather, it increased at a specific bias. However, the responsivity decreased sharply when the absorbed dose exceeded 400 Gy. For example, at the gate bias lower than the threshold by 15 V, the responsivity slightly decreased from 6.24 A/W to 5.9 A/W after 200 Gy of gamma radiation. However, after 400 Gy of gamma irradiation, the responsivity decreased from 6.24 A/W to 1.82 A/W. This implies that the gamma irradiation has a significant effect on the responsivity of the MoS_2_ phototransistor.

We also characterized the variation of responsivity according to the incident optical power density for the MoS_2_ phototransistor irradiated with gamma rays as shown in Fig. 2b. Before the gamma ray irradiation, as an incident optical power density increases, the responsivity increases at a lower incident power and gradually saturates due to saturation of traps. The similar trend was observed in the device irradiated by 400 Gy gamma rays, but the overall responsivity decreases. When 800 Gy gamma rays were irradiated, the responsivity saturated at lower optical power density and decreases as the optical power density further increases.

**Figure 3:**
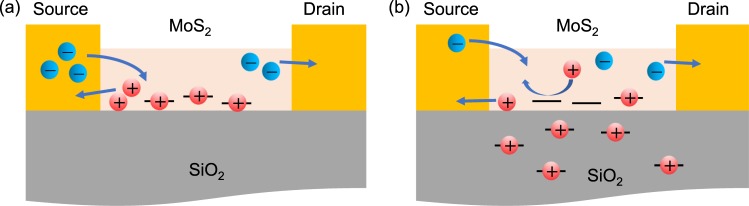
(**a**) Schematic of the photogating effect of the MoS_2_ phototransistor. (**b**) Schematic of positive oxide charge effect occurred by gamma irradiation.

The responsivity of the MoS_2_ phototransistor is obtained because the electrons are supplied into the channel from the source by the photogenerated holes trapped at the interface between MoS_2_ and SiO_2_^13,23,39^, as depicted in Fig. 3a. Therefore, we posit that the gamma rays affected the SiO_2_, which is the gate dielectric of the MoS_2_ phototransistor. Gamma radiation has two major effects on SiO_2_^40,41^. The first effect is ionization, caused by Compton scattering and the photoelectric effect. The ionization effect separates the electrons from the atoms. These separated electrons drift away from the SiO_2_ to a positive electrode. Meanwhile, holes are trapped in trivalent-silicon traps or nonbridging oxygen traps and remain in the oxide. The second effect is atomic displacement. When an atom collides with a gamma ray with large kinetic energy, it is pushed out of its initial location. The pushed atoms cause additional atomic movements, resulting in a cluster of defects. These two effects of gamma radiation significantly increase the positive oxide charge in the SiO_2_ after gamma irradiation. Consequently, the positive oxide charges reduce the probability of photogenerated holes being trapped at the interface between MoS_2_ and SiO_2_, as illustrated in Fig. 3b and the responsivity of the MoS_2_ phototransistor decreases. Therefore, the MoS_2_ phototransistors directly irradiated by the high-energy gamma rays reduced in responsivity significantly.

One may think that by reducing the thickness of SiO_2_, the chance of gamma radiation damage and consequently the responsivity degradation could be mitigated. However, the positive charges spread in the SiO_2_ will not equally contribute to the decrease in the responsivity, and the charges close to the interface between MoS_2_ and SiO_2_ will have a greater impact. It will be able to define the effective distance from the interface for convenience, and the positive charges within this range mostly disturb the trappings of the photogenerated holes in MoS_2_. Because the SiO_2_ is required to be thicker than the effective distance to prevent a gate leakage, the thickness of SiO_2_ will not have a significant effect on the performance deterioration due to gamma ray.

**Figure 4:**
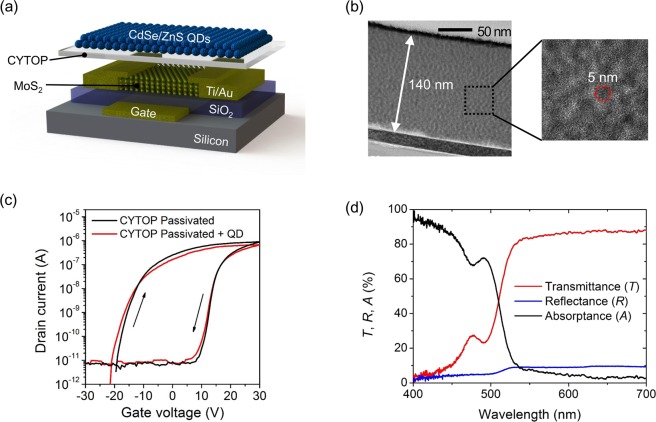
(**a**) Schematic of the MoS_2_ phototransistor with CYTOP/QD layer. (**b**) TEM image of the QD layer. (**c**) Transfer curves of MoS_2_ phototransistor with CYTOP layer or CYTOP/QD layer. (**d**) Transmittance, reflectance, and absorptance of CYTOP/QD layer.

To reduce these degradation of device characteristics by the gamma irradiation, we passivated the MoS_2_ phototransistor with the CYTOP and CdSe/ZnS QD layer. The fabricated MoS_2_ phototransistor is depicted in Fig. 4a. The CYTOP layer was spin-coated to a thickness of 1 μm on top of MoS_2_; subsequently, the QD layer was passivated by the drop-casting method. The resulting thickness of the QD layer was approximately 140 nm as shown in Fig. 4b. The deposition of the CYTOP layer on the MoS_2_ is to prevent the QD from directly affecting the MoS_2_ channel. In a study about QDs and the MoS_2_ interface by Dominik Kufer *et al*.^42^, when the QD layer is directly deposited on MoS_2_, the ON/OFF ratio and subthreshold swing are significantly reduced. The modulation loss is associated with Fermi-level pinning. When MoS_2_, which has many defects on its surface, directly contacts the QD layer, a large density of localized states occurs between them. It fixes the Fermi-level, thus decreasing the modulation via the gate bias. Meanwhile, when the CYTOP layer is inserted between the MoS_2_ and QD layer, the transfer characteristics are almost unchanged as shown in Fig. 4c. This is because the CYTOP layer prevents the occurrence of a localized state that causes Fermi-level pinning between MoS_2_ and the QD layer. Figure 4d shows the measured transmittance (*T*) and reflectance (*R*) of the identical CYTOP/QD layer fabricated on glass. The spectral absorptance (*A*) is also shown in the figure. It was calculated by the equation: *A* = 1 − (*R* + *T*), assuming that stray light is negligible. The CYTOP/QD layer absorbs the light with wavelengths shorter than 520 nm and this absorption is mostly contributed by the QD because the CYTOP is transparent in all visible region. At the wavelength of 466 nm, which is the laser wavelength used in the experiment, the QD absorbs 73.7% of incoming light and the transmitted 21.5% is incident on the underlying MoS_2_ phototransistor.

**Figure 5:**
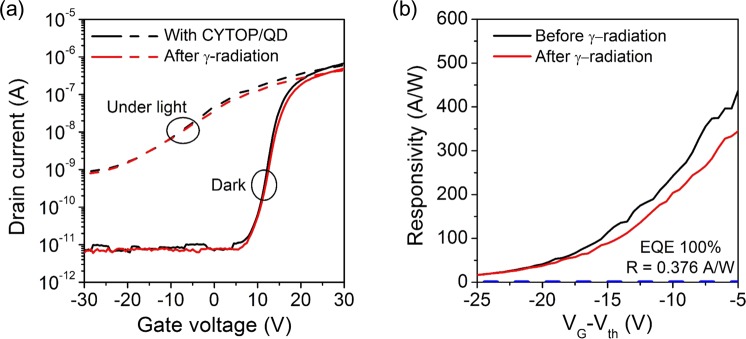
(**a**) Transfer curves of MoS_2_ phototransistor with CYTOP/QD layer measured before and after gamma irradiation. (**b**) Responsivity of MoS_2_ phototransistor with CYTOP/QD layer measured before and after gamma irradiation.

The radiation test of the MoS_2_ phototransistor with CYTOP/QD layer was performed under the same conditions as the bare MoS_2_ phototransistor. Figure 5a shows the transfer curves of the MoS_2_ phototransistor with CYTOP/QD layer measured before and after gamma irradiation. The electrical characteristics of the MoS_2_ phototransistor with CYTOP/QD layer were extracted from the solid line in Fig. 5a by the same methods as above. The threshold voltage of the MoS_2_ phototransistor with CYTOP/QD layer was shifted from 13.9 V to 14.9 V after gamma irradiation. The field effect mobility decreased from 2.46 cm^2^/V∙s to 2.23 cm^2^/V∙s after gamma irradiation. The subthreshold swing increased from 1.4 V/dec to 1.6 V/dec after gamma irradiation. The electrical characteristics did not show any significant difference between before and after gamma irradiation. In addition, unlike the bare MoS_2_ phototransistor, the transfer curve of the MoS_2_ phototransistor with CYTOP/QD layer measured at 2 mW/cm^2^ optical power density (dashed line in Fig. 5a) shows little change after gamma irradiation. Figure 5b shows the responsivity of the MoS_2_ phototransistor with CYTOP/QD layer extracted from the dashed line in Fig. 5a. As shown in Fig. 5b, the responsivities for all gate voltages were larger than 0.376 A/W corresponding to 100% of external quantum efficiency. Thus, it can be seen that the CYTOP/QD layer does not limit the detecting range of the MoS_2_ phototransistor. More importantly, the responsivity was decreased by only 21.9% (from 114 A/W to 88.7 A/W) after gamma radiation at the gate bias lower than the threshold by 15 V. This indicates that the CYTOP/QD layer can effectively prevent the responsivity degradation of the MoS_2_ phototransistor. This is because the QD with a high atomic number was used as the protection layer. The QD with a high atomic number has a high probability of colliding with the gamma rays, which causes the incident gamma rays to scatter and subsequently the scattered gamma rays reaches the MoS_2_ phototransistor. Because the scattered gamma rays have relatively low energy, they induce small positive oxide charges, resulting in no significant decrease in the responsivity.

**Figure 6:**
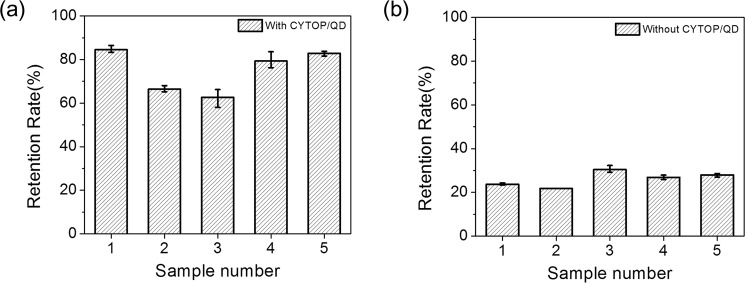
Bar graph of retention rate of (**a**) MoS_2_ phototransistors with CYTOP/QD layer after gamma irradiation and (**b**) bare MoS_2_ phototransistors after gamma irradiation.

To confirm the consistency of the QD layer effect, we fabricated several devices and performed a gamma ray degradation test similarly. Figure 6 is a bar graph showing how the responsivity of the MoS_2_ phototransistors is maintained after the gamma ray degradation test. Since the photoresponse gains are affected by the gate voltages, the responsivities should be compared at the gate biases that are negatively shifted by the same voltage from the threshold voltage of each device. The responsivities were estimated at the gate biases that are lower than each threshold voltages by 14, 15 and 16 V, and their average values were shown in Fig. 6. When the MoS_2_ phototransistors were passivated by the CYTOP/QD layer (Fig. 6a), the retention rate of responsivity averaged at 75.16% after gamma irradiation. For the bare MoS_2_ phototransistors (Fig. 6b), the average retention rate of responsivity was 26.17% after gamma irradiation. These results show that the MoS_2_ phototransistor with QD layer has statistically better radiation hardness.

## Conclusions

To summarize, we studied the effect of gamma rays on the MoS_2_ phototransistor and its prevention mechanism. When the amount of gamma ray irradiated to MoS_2_ phototransistor reached a certain level, it was confirmed that the electrical characteristics and the responsivity were significantly degraded. These results show that the gamma rays have a significant influence on both MoS_2_ channel and the gate dielectric. In the MoS_2_ irradiated by the gamma ray, atomic displacement is induced, which interferes with the movement of the carrier, thereby reducing on-current and field effect mobility. The SiO_2_ used as the gate dielectric has many positive oxide charges after gamma irradiation. These positive oxide charges reduce the responsivity by preventing holes that increase the optical gain from being trapped at the interface of MoS_2_ and SiO_2_. We used the CdSe/ZnS quantum dots in a protection layer to prevent the responsivity degradation of the MoS_2_ phototransistor due to gamma irradiation. The irradiated gamma rays are scattered owing to the high atomic number QD and rarely cause positive oxide charges. Therefore, the MoS_2_ phototransistor with QD layer can maintain its responsivity better than the bare MoS_2_ phototransistor after gamma irradiation. Through this experiment, we confirmed that the MoS_2_ phototransistor with QD such as CdSe/ZnS could be used in harsh environments such as space and nuclear industries. In addition, QD protection layers can be applied to other devices, such as solar cells and photodetectors, in order to improve their radiation hardness.

## Methods

### MoS_2_ phototransistor fabrication

On the silicon substrate, gate electrodes are patterned using photolithography and Ti/Au (5/80 nm) were deposited on the patterns using an e-beam evaporator. SiO_2_ was deposited as the gate electric at 300 °C using the plasma-enhanced chemical vapor deposition (PECVD) method. MoS_2_ flakes were mechanically exfoliated from bulk MoS_2_ crystals (Graphene Supermarket, USA) and transferred onto the gate dielectric using the conventional Scotch-tape method. The source and drain electrodes were patterned on the MoS_2_ flakes and Ti/Au (5/80 nm) were deposited using an e-beam evaporator.

### CYTOP and CdSe/ZnS QD layer deposition

CYTOP (Asahi Glass, Japan) was spincoated on the MoS_2_ at a rotation speed of 2000 rpm and cured at 180 °C for 60 min. The expected thickness of the CYTOP layer was approximately 1 μm. The CYTOP layer over the source and drain electrodes was etched using plasma etching. The CdSe/ZnS quantum dot (Sigma Aldrich, USA) layer was deposited on the CYTOP layer by dropcasting the QD dispersion and cured at 60 °C for 30 min. The QD concentration used was 5 mg/mL in toluene. This process was repeated two more times. The resulting thickness of the QD layer was approximately 140 nm.

### Gamma ray irradiation

The gamma ray irradiation was performed using Co-60 source and the total absorbed dose was varied from 200 Gy to 800 Gy.

### Device characterization

JEM-2100F (JEOL, USA) was used to obtain the TEM image of the CdSe/ZnS quantum dot layer. Current–voltage (${I}_{D}-{V}_{{GS}}$) measurements were performed using a dual-channel source meter (Keithley 2614B) at room temperature. The responsivity of the MoS_2_ phototransistor was measured under illumination with 2 mW/cm^2^ power density at 466 nm (Civillaser).

## Supplementary information

supplementary information

## References

[1] Radisavljevic B, Radenovic A, Brivio J, Giacometii V, Kis A (2011). Single-layer MoS_2_ transistors. Nat. Nanotechnol..

[2] Wang QH, Kalantar-Zadeh K, Kis A, Coleman JN, Strano MSStrano (2012). Electronics and optoelectronics of two-dimensional transition metal dichalcogneides. Nat. Nanotechnol..

[3] Akinwande D, Petrone N, Hone J (2014). Two-dimensional flexible nanoelectronics. Nat. Commun..

[4] Ruitao Lv JA (2015). Transition Metal Dichalcogenides and Beyond: Synthesis, Properties, and Applications of Single- and Few-Layer Nanosheets. Acc. Chem. Res..

[5] Duan X, Wang C, Pan A, Yu R, Duan X (2015). Two-dimensional transition metal dichalcogenides as atomically thin semiconductors: opportunities and challenges. Chem. Soc. Rev..

[6] Jariwala D, Sangwan VK, Lauhon LJ, Marks TJ, Hersam MC (2014). Emerging Device Applications for Semiconducting Two-Dimensional Transition Metal Dichalcogenides. ACS Nano..

[7] Kam KK, Parkinson BA (1982). Detailed photocurrent spectroscopy of the semiconducting group VIB Transition Metal dichalcogenides. J. Phys. Chem..

[8] Mak KF, Lee C, Hone J, Shan J, Heinz TF (2010). Atomically Thin MoS_2_: A New Direct-Gap Semiconductor. Phys. Rev. Lett..

[9] Tsai D-S (2013). Few-Layer MoS_2_ with High Broadband Photogain and Fast Optical Switching for Use in Harsh Environments. ACS Nano.

[10] Bernardi M, Palummo M, Grossman. JC (2013). Extraordinary Sunlight Absorption and One Nanometer Thick Photovoltaics Using Two-Dimensional Monolayer Materials. Nano Lett..

[11] Beal AR, Hughes HP (1979). Kramers-Kronig analysis of the reflectivity spectra of 2H-MoS_2_, 2H-MoSe_2_ and 2H-MoTe_2_. J. Phys. C: Solid Phys..

[12] Xu H (2014). High Responsivity and Gate Tunable Graphene-MoS2 Hybrid Phototransistor. Small.

[13] Park J, Park Y, Yoo G, Heo J (2017). Bias-dependent photoresponsivity of multi-layer MoS_2_ phototransistors. Nanoscale Res. Lett..

[14] Kwak JY, Hwang J, Calderon B, Alsalman H, Spencer MG (2016). Long wavelength optical response of graphene-MoS_2_ heterojunction. Appl. Phys. Lett..

[15] Hugo Henck D (2016). Electrolytic phototransistor based on graphene-MoS_2_ van der waals p-n heterojunction with tunable photoresponse. Appl. Phys. Lett..

[16] Yu SH (2014). Dye-Sensitized MoS_2_ Photodetector with Enhanced Spectral Photoresponse. ACS Nano..

[17] Huo N (2014). Novel and Enhanced Optoelectronics Performances of Multilayer MoS_2_-WS_2_ Heterostructure Transistors. Adv. Funct. Mater.

[18] Kwon J (2015). Giant Photoamplification in Indirect‐Bandgap Multilayer MoS_2_ Phototransistors with Local Bottom‐Gate Structures. Adv. Mat..

[19] Choi W (2012). High-Detectivity Multilayer MoS_2_ Phototransistors with Spectral Response from Ultraviolet to Infrared. Adv. Mater..

[20] Schornbaum JB (2014). Epitaxial Growth of PbSe Quantum Dots on MoS_2_ Nanosheets and their Near-Infrared Photoresponse. Adv. Funct. Mater.

[21] Kufer D (2015). Hybrid 2D-0D MoS_2_-PbS Quantum Dot Photodetectors. Adv. Mater..

[22] Chen C (2015). Highly responsive MoS_2_ photodetectors enhanced by graphene quantum dots. Sci. Rep..

[23] Zhang W (2014). Ultrahigh-Gain Photodetectors Based on Atomically Thin Graphene-MoS_2_ Heterostructures. Sci. Rep..

[24] Yoo G (2017). Flexible and Wavelength-Selective MoS2 Phototransistors with Monolithically Integrated Transmission Color Filters. Sci. Rep..

[25] Lee S, Park Y, Yoo G, Heo J (2017). Wavelength-selective enhancement of photo-responsivity in metal-gated multi-layer MoS_2_ phototransistors. Appl. Phys. Lett..

[26] Zhang Y (2016). *In Situ* Fabrication of Vertical Multilayered MoS_2_/Si Homotype Heterojunction for High‐Speed Visible–Near‐Infrared Photodetectors. Small.

[27] Kufer D, Konstantatos G (2015). Highly Sensitive, Encapsulated MoS_2_ Photodetector with Gate Controllable Gain and Speed. Nano Lett..

[28] Li X (2015). A self-powered graphene–MoS_2_ hybrid phototransistor with fast response rate and high on–off ratio. CARBON.

[29] Fazel R (2009). Exposure to Low-Dose Ionizing Radiation from Medical Imaging Procedures. N Engl J Med.

[30] Sultana A (2007). Systematic review, including meta-analyses, on the management of locally advanced pancreatic cancer using radiation/combined modality therapy. BJC.

[31] Kader AA (1986). Potential Applications of Ionizing Radiation in postharvest Handling of Fresh Fruit and vegetables. Food Tech..

[32] Jianlong W, Jiazhuo W (2007). Application of radiation technology to sewage sludge processing: A review. J. Haz. Mat..

[33] Koenig TR, Wolff D, Mettler FA, Wagner LK (2001). Skin Injuries from Fluoroscopically Guided Procedures: Part 1, Characteristics of Radiation. Injury. AJR.

[34] Sutherland BM, Bennett PV, Sidorkina O, Laval J (2000). Clustered DNA damages induced in isolated DNA and in human cells by low doses of ionizing radiation. PNAS..

[35] Reichmanis E, Frank CW, O’Donnell JH, Hill DJT (1993). Radiation Effects on Polymeric Materials: A Brief Overview. ACS S. S..

[36] Polyakov AY (2013). Radiation effects in GaN materials and devices. J. Mater. Chem. C..

[37] Algara-Siller G, Kurasch S, Sedighi M, Lehtinen O, Kaiser U (2013). The pristine atomic structure of MoS_2_ monolayer protected from electron radiation damage by graphene. Appl. Phys. Lett..

[38] Recep Z (2013). Control of Radiation Damage in MoS_2_ by Graphene Encapsulation. ACS Nano.

[39] Furchi MM, Polyushkin DK, Pospischil A, Mueller T (2014). Mechanisms of Photoconductivity in Atomically Thin MoS_2_. Nano Lett..

[40] Dimitrijev S, Golubovic S, Zupac D, Pejovic M, Stojadinovic N (1989). Analysis of gamma-Radiation Induced Instability Mechanisms in CMOS Transistors. Solid-St. Electron..

[41] Nikolic D, Stankovic K, Timotijevic L, Rajovic Z, Vujisic M (2013). Comparative Study of Gamma Radiation Effects on Solar Cells, Photodiodes, and Phototransistors. IJP..

[42] Kufer D, Lasanta T, Bernechea M, Koppens FHL, Konstantatos G (2016). Interface Engineering in Hybrid Quantum Dot-2D Phototransistors. ACS Photonics.

