# Supplementary material for: CdSe/ZnS quantum dot encapsulated MoS2 phototransistor for enhanced radiation hardness
Source: Sci Rep. 2019 Feb 5;9:1411. doi: 10.1038/s41598-018-37902-y (PMC6363733; doi:10.1038/s41598-018-37902-y)
Supplement: Supplementary file 1 — supplementary information [file 41598_2018_37902_MOESM1_ESM.docx]

**Supplementary Information**

CdSe/ZnS quantum dot encapsulated MoS_2_ phototransistor for enhanced radiation hardness

Jinwu Park^1^, Geonwook Yoo^2^, Junseok Heo^1*^

*^1^Department of Electrical and Computer Engineering, Ajou University, Suwon 16499, South Korea*

*^2^School of Electronic Engineering, Soongsil University, Seoul 06938, South Korea*

^*^Corresponding authors: jsheo@ajou.ac.kr

**1. Degradation test of MoS_2_ phototransistor at 20 kGy dose of gamma ray.**


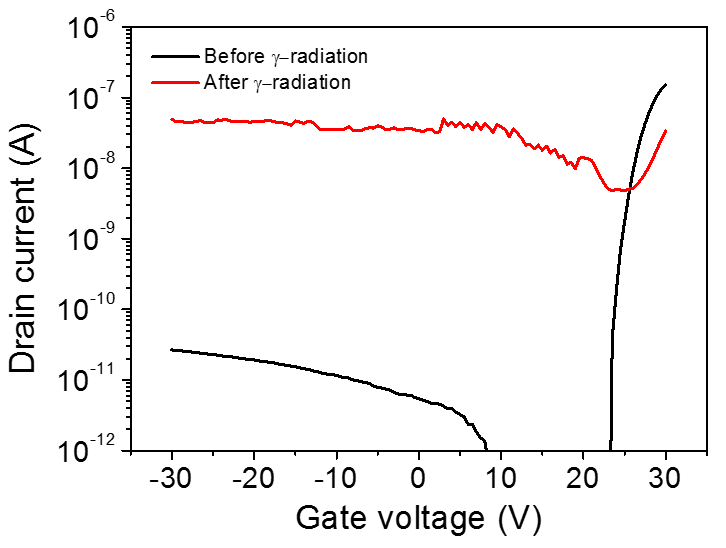


Fig. S2 Transfer characteristics of MoS_2_ phototransistor before and after 20 kGy of gamma ray.

The high energy gamma ray irradiation was performed using Co-60 source and the total absorbed dose was 20 kGy. The electrical characteristics of MoS_2_ phototransistor are completely impaired after gamma irradiation.
